# Cancer-testis gene expression is associated with the *methylenetetrahydrofolate reductase* 677 C>T polymorphism in non-small cell lung carcinoma

**DOI:** 10.1186/1471-2350-14-97

**Published:** 2013-09-24

**Authors:** Kerem M Senses, Mithat Gonen, Ahmet R Barutcu, Zeynep Kalaylioglu, Murat Isbilen, Ozlen Konu, Yao T Chen, Nasser K Altorki, Ali O Gure

**Affiliations:** 1 Department of Molecular Biology and Genetics, Bilkent University, Ankara, Turkey; 2 Department of Epidemiology and Biostatistics, Memorial Sloan Kettering Cancer Center, New York, NY, USA; 3 Department of Statistics, Middle East Technical University, Ankara, Turkey; 4 Department of Pathology, Weill Medical College of Cornell University, New York, NY, USA; 5 Department of Cardiothoracic Surgery, Weill Medical College of Cornell University, New York, NY, USA; 6 Department of Cell and Developmental Biology, University of Massachusetts Medical School, Worcester, MA, USA

**Keywords:** Cancer-testis genes, 1-carbon pathway, Adomet, DNA methylation

## Abstract

**Background:**

Tumor-specific, coordinate expression of cancer-testis (CT) genes, mapping to the X chromosome, is observed in more than 60% of non-small cell lung cancer (NSCLC) patients. Although CT gene expression has been unequivocally related to DNA demethylation of promoter regions, the underlying mechanism leading to loss of promoter methylation remains elusive. Polymorphisms of enzymes within the 1-carbon pathway have been shown to affect S-adenosyl methionine (SAM) production, which is the sole methyl donor in the cell. Allelic variants of several enzymes within this pathway have been associated with altered SAM levels either directly, or indirectly as reflected by altered levels of SAH and Homocysteine levels, and altered levels of DNA methylation. We, therefore, asked whether the five most commonly occurring polymorphisms in four of the enzymes in the 1-carbon pathway associated with CT gene expression status in patients with NSCLC.

**Methods:**

Fifty patients among a cohort of 763 with NSCLC were selected based on CT gene expression status and typed for five polymorphisms in four genes known to affect SAM generation by allele specific q-PCR and RFLP.

**Results:**

We identified a significant association between CT gene expression and the *MTHFR* 677 CC genotype, as well as the C allele of the SNP, in this cohort of patients. Multivariate analysis revealed that the genotype and allele strongly associate with CT gene expression, independent of potential confounders.

**Conclusions:**

Although CT gene expression is associated with DNA demethylation, in NSCLC, our data suggests this is unlikely to be the result of decreased MTHFR function.

## Background

Cancer-testis (CT), or cancer-germline genes, currently with more than 100 members, are distinctly expressed in cancer, germline and trophoblast cells but not in other normal tissues in the adult. Most CT genes constitute multigene families organized in clusters along the X chromosome. Members within a family are highly homologous, however, no conservation of sequence exists between families [1]. Despite the lack of sequence similarity (including promoters), re-expression of almost all CT genes in tumors correlates with the demethylation of their promoters that occurs in parallel to a genome-wide demethylation event, primarily affecting repeat regions [2]. The mechanisms leading to CT gene promoter demethylation in cancer are unknown. Increased BORIS expression has been associated with upregulated CT gene expression [3,4], but the protein is likely not the sole responsible factor in this event. Histone acetylation has also been shown to facilitate CT gene expression, primarily when it associates with DNA demethylation [5].

As most CT gene products are highly antigenic they have been utilized in clinical trials based on immunotherapeutic approaches targeting these antigens [6]. Since patient eligibility for CT targeting immunotherapy requires that the tumor express CT genes, it is important to know whether CT gene expression can be induced. It is expected that any approach leading to CT gene expression should also result in the demethylation of their promoters.

Production of the sole methyl donor in the cell, S-adenosylmethionine (SAM), depends on the efficient utilization of folate, by the 1-carbon pathway. Several enzymes in this pathway contain common polymorphic variants that reduce the efficiency of the enzyme and thus, the rate of SAM production. Hypomorphic alleles of four of these enzymes (methylenetetrahydrofolate reductase (*MTHFR*), methionine synthase reductase (*MTRR*), methionine synthase (*MTR*)*,* and reduced folate carrier (*RFC*)), have been associated with cellular under-utilization of folate and homocysteine, increased DNA hypomethylation, and decreased CpG methylation [7-11]. More recently, the hypomorphic 677 T allele of *MTHFR*, has been associataed with the expression of *MAGE-A1*, a CT gene, in glioblastoma multiforme [12]. Others, however, could not reproduce these findings in ovarian carcinoma [13]. In the present study we asked if polymorphisms of the 1-carbon pathway enzymes associate with CT gene expression in non-small cell lung cancer (NSCLC) patients. Our results show a strong association between the *MTHFR*677 CC genotype as well as the *MTHFR* 677 C allele and CT gene expression independent of age, sex, histology, and tumor stage.

## Methods

### Patients and tumor material

Tumor samples obtained from patients undergoing curative surgical resection for primary NSCLC at the Department of Cardio-Thoracic Surgery, Weill Medical College of Cornell University, from 1991 to July 2005 were analyzed in this study. Informed consent was obtained from all patients. The study was approved by the Institutional Review Board of Weill Medical College of Cornell University. Fifty tumor samples were selected solely based on CT gene expression from 763 samples that had been evaluated for the presence of transcripts from up to 9 CT genes (*NY-ESO-1, LAGE-1, MAGE-A1, MAGE-A3, MAGE-A4, MAGE-A10, CT-7, SSX2,* and *SSX4*), by semi-quantitative PCR, as described previously [14]. Twenty one samples with CT expression in at least 4 of the 9 CT genes tested, with strong expression in at least one gene, constituted the CT (+) group. Twenty-nine samples with no CT expression in any of the CT genes tested (with a minimum of 5 CT genes tested) were selected as CT (-) tumors for this study. CT gene expression was determined as strong (+++), intermediate (++), weak (+ or +/-), or none (-) as previously described [14], and is shown in Additional file 1: Table S1.

### DNA analysis

Genomic DNA extracted from tumor tissues were genotyped using pre-designed 5’-nuclease TaqMan SNP genotyping assays (Applied Biosystems, Foster City, CA) using a Stratagene Mx3005P instrument according to the manufacturer’s instructions. The SNPs typed and their reference IDs were: *MTHFR* 677 C> T (rs1801133), *MTHFR* 1298 A>C (rs1801131), *MTR* 2756 A>G (rs1805087), and *MTRR* 66 A>G (rs1801394). Nested PCR-RFLP was used to type the *RFC* 80 G>A (rs1051266) polymorphism for which the first round PCR conditions were previously described [10]. Nested PCR primers were: 5’- AGCCGTAGAAGCAAAGGTAGC-3’ and 5’-AGCGTCACCTTCGTCCCCTC-3’. PCR was performed using DyNAzyme™ II Hot Start DNA Polymerase (Finnzymes, Keilaranta, Finland). PCR conditions were: 10’ activation at 94°C, followed by 35 cycles of 94°C, 62°C and 72°C; 30” each, with a final 72°C, 7’ extension. HinP1I (New England Biolabs, Hertfordshire, UK) digested PCR products were analyzed as described previously [10]. All analyses were repeated at least twice.

Genotypes for all polymorphisms were determined successfully in all cases (Additional file 2: Table S2). Genotype distributions did not deviate from Hardy-Weinberg equilibrium (Additional file 3: Table S3). Minor allele frequencies for individual loci were: 40% for *MTHFR* 677 C > T*,* 26% for *MTHFR* 1298 A > C*,* 14% for *MTR* 2756 A > G*,* 54% for *MTRR* 66*,* and 42% for *RFC* 80 G > A. *MTHFR* genotypes were not independently distributed across the 2 loci. The major 677C allele was in linkage disequilibrium with the minor 1298C allele (D’ = 0.99, r^2^ = 0.23) [15].

### *In silico* association analysis

Paired datasets, GSE14471 and GSE15714, containing gene expression and SNP genotyping data, respectively, from 111 pediatric acute myeloid leukemia samples (of which 109 were typed successfully), were analyzed for an association between CT gene expression and *MTHFR* 677 genotype distribution [16]. A principal component analysis using 44 probesets corresponding to 9 CT gene families was performed for the expression dataset. The first principal component, explaining 0.48 of variance for CT gene expression was used to generate groups representing samples with low, intermediate, and high CT gene expression by K means clustering using a customized R code [17]. Optimum number of clusters according to Elbow criterion was determined as five. Therefore, five initial cluster centers were placed equally distant from each other where the first and last centers represented the minimum and maximum values of PC1, respectively. Centers were iteratively updated based on the median value of the reassigned cluster members until no change in cluster membership took place. The five clusters were regrouped into three representing low (clusters 1 & 2), intermediate (cluster 3), and high CT gene expression (clusters 4 & 5).

### Statistical analysis

To analyze the association between 1-carbon pathway enzyme polymorphisms and CT gene expression, the genotype distributions were compared in CT (+) and CT (-) tumors by Pearson’s Chi-Square (2 degrees of freedom) or Fisher’s exact tests. Odds ratios (OR) were estimated by multivariate logistic regression. To evaluate whether CT gene expression was related to sex, smoking status, tumor size, and disease stage, Fisher's exact test or Chi-square tests were used. Race information was available for only 29 patients of which 25 were non-Hispanic white, one was a non-Hispanic black, and 3 were of mixed race, and was not included in statistical analyses. All statistical tests were two-sided with a 5% type I error rate, unless indicated otherwise, and were carried out using SAS (version 9.3) software (SAS Institute, Cary, NC). *P* < 0.05 was considered statistically significant.

## Results

Demographics and clinical characteristics of patients and their distribution within CT (+) and (-) groups are shown in Table 1 and Additional file 1: Table S1. Tumors with non-squamous cell carcinoma histology and earlier tumor stage (T stage) showed lower CT gene expression, similar to what has been reported previously [14]. Distribution of individual genotypes among CT (+) and (-) tumors are shown in Table 2 and Additional file 2: Table S2. A significant association between the *MTHFR* 677CC genotype and CT expression was observed (*P* = 0.03). CT expression was not related to any other genotype tested. A multivariate logistic regression analysis (MVA) of CT gene expression that included the *MTHFR* 677 genotype distribution, age, sex, histology and T stage revealed that the *MTHFR* 677 genotype and histology were independent predictors of CT gene expression in this cohort (Table 3). The *MTHFR* 677 SNP was found to be associated with CT gene expression when analyzed on a per allele basis, controlling for confounding factors, while other markers were not (Table 4). We performed an *in silico* association analysis for CT expression and the *MTHFR* 677 genotype using two datasets derived from childhood acute myeloid leukemia (AML) where both gene expression and SNP genotyping data were available [16]. This analysis, however, did not reveal a statistically significant association between these two parameters (Table 5 and Additional file 4: Figure S1).

**Table 1:** Demographics and clinical characteristics

		**CT (+) patients (n=21)**	**CT (-) patients (n=29)**	P*
Age	>60	12	19	0.74
	<=60	6	7	
	Unknown^$^	3	3	
Sex	Male	10	12	0.76
	Female	8	14	
	Unknown	3	3	
Smoking history	No	1	5	0.37
	Yes	15	18	
	Unknown	5	6	
Histology	SQCC^#^	8	2	0.002
	non-SQCC	7	24	
	Unknown	6	3	
Pathological tumor size	>3 cm	10	8	0.21
	<=3 cm	8	17	
	Unknown	3	4	
T stage	1	5	14	0.007
	2	8	12	
	3	3	0	
	4	2	0	
	Unknown	3	3	
TNM stage (Pathologic stage of primary tumor)	I	9	18	0.37
	II	5	3	
	III	4	5	
	IV	0	0	
	Unknown	3	3	

* Chi-square (Fisher's exact test, two sided) or chi-square test for trend; ^#^*SQCC* Squamous cell carcinoma; ^$^patients with missing clinical data were not included in statistical analyses.

**Table 2:** Distribution of individual genotypes among CT (+) and CT (-) tumors

***Polymorphism***	***Genotype***	***CT (+) Tumors, n (%)***	***CT (-) Tumors, n (%)***	***χ2***	P*
*MTHFR* 677 C>T (rs1801133)	CC	13 (62*%*)	7 (24%)	7.30	0.03
	CT	5 (24%)	15 (52%)		
	TT	3 (14%)	7 (24%)		
*MTHFR* 1298 A>C (rs1801131)	AA	13 (62%)	14 (48%)	0.91	0.63
	AC	7 (33%)	13 (45%)		
	CC	1 (5%)	2 (7%)		
*MTR* 2756 A>G (rs1805087)	AA	14 (67%)	22 (76%)	0.51	0.53**
	AG	7 (33%)	7 (24%)		
	GG	0 (0%)	0 (0%)		
*MTRR* 66 A>G (rs1801394)	AA	4 (19%)	9 (31%)	0.92	0.63
	AG	10 (48%)	12 (41%)		
	GG	7 (33%)	8 (28%)		
*RFC* 80 G>A (rs1051266)	GG	6 (29%)	12 (41%)	1.90	0.39
	GA	9 (43%)	13 (45%)		
	AA	6 (29%)	4 (14%)		

* Chi-square (2 degrees of freedom); ** Fisher’s exact test, two sided.

**Table 3:** Multivariate analysis of CT gene expression with MTHFR 677 genotypes

***Parameter***		***OR***	***95% CI***	P*
*MTHFR* 677 C>T (rs1801133)	CC	32.33	2.42-431.52	0.003
	CT/TT	1**		
Age^$^		1.03	0.94-1.12	0.69
*Sex(female vs. male)*		0.28^*‡*^	0.02-3.86	0.52
*Histology*	SQCC^#^	18.46	1.19-284.49	0.04
	non-SQCC	1**		
*T Stage*	T1	0.31	0.03-3.56	0.53
	T2, 3, 4	1**		

* Computed from a logistic regression model using the EXACT option of PROC LOGISTIC in SAS to account for the small data set; ^$^continuous variable. ^#^*SQCC* Squamous cell carcinoma; ^*‡*^reference = male; **reference group.

**Table 4:** Multivariate logistic regression modeling of association between 1-carbon enzyme alleles and CT gene expression

***Polymorphism***	***OR***^$^	***95% CI***	P*
*MTHFR* 677 (rs1801133)	13.18	1.96-88.5	0.004
*MTHFR* 1298 (rs1801131)	0.56	0.15-2.09	0.53
*MTR* 2756 (rs1805087)	0.81	0.14-4.75	1
*MTRR* 66 (rs1801394)	0.67	0.22-2.00	0.52
*RFC* 80 (rs1051266)	0.7	0.24-2.07	0.62

^$^ Based on number of major alleles, adjusted for age, sex, histology and t stage; * computed using the EXACT option of PROC LOGISTIC in SAS to account for the small data set.

**Table 5:** ***In silico *correlation of CT gene expression with *MTHFR *677 genotypes in acute Myeloid Leukemia**

**CT gene expression clusters**	***MTHFR *677 C>T (rs1801133)**			***P *(chi-square)**
	**CC**	**CT/TT**	Untyped*	
High	15	16	0	0.17
Intermediate	20	13	0	
Low	29	16	2	

* Untyped samples were not included in analysis.

## Discussion

Among the five markers analyzed in this study, we find a strong association between the major *MTHFR* 677 CC genotype, as well as the *MTHFR* 677 C allele and CT gene expression in lung cancer. This contrasts with earlier studies where the minor allele of this SNP was associated with decreased SAM production, decreased methylation levels and decreased *MAGE-A1* expression [12]. Although our analysis included only 7% of patients within a large cohort with the highest and lowest amount of CT gene expression, we don’t think this is a reason for bias, as the distribution of the 1-carbon pathway genotypes of our samples are similar to those where much larger lung cancer patient cohorts were evaluated [18-20]. Tumors of squamous cell histology were previously identified as showing more frequent and stronger CT gene expression; however, MVA shows that the association between *MTHFR* 677 CC genotype or the C allele of the same polymorphism and CT gene expression is independent of histology. On the other hand, tumor type is known to affect CT gene expression rates, as some blood-derived tumors and cancers originating from the kidney rarely express CT genes [21]. In this line, one reason for our inability to replicate our q-PCR based results *in silico* might be related to the fact that AML is not a tumor with strong CT expression and thus, the K-means based classification of this tumor is somewhat artificial. Therefore, a similar analysis with datasets ideally derived from lung cancer might reveal associations not identified in this study.

We calculated the sample size that would give us 80% power to detect a significant association between polymorphisms other than *MTHFR* 677 and CT gene expression using the observed effect sizes in this study as true values. We found that at least 250 patients would be required to find one more polymorphism significant. Therefore, analysis of larger cohorts might reveal additional associations as well as compound effects of SNPs within the 1-carbon pathway enzymes on CT gene expression. Models to test for such effects were not computed in this study due to the limited sample size.

Although decreased SAM levels might be expected to result in DNA demethylation, the exact SAM concentration threshold required for gene re-expression might be affected by various other parameters not tested in this study. A candidate is thymidylate synthase (TS) whose levels are known to fluctuate widely in cancer and which can inhibit MTHFR activity [22]. CT gene expression is associated with larger tumors and advanced stage [14]. If this is to be taken as a sign of increased proliferation, it would imply increased TS activity, and thus, possibly suppressed MTHFR, which in turn could affect CT gene expression. On the other hand, increased SAM production might indirectly inhibit methylation reactions via methylthioadenosine (MTA), a nucleoside produced from SAM through the polyamine biosynthetic pathway. MTA can strongly inhibit H3K4 methylation, possibly by inhibiting Set1 methyltransferase, which could in turn result in repressed CT gene expression [23-25]. Future studies are necessary to explain which of these primarily affect methylation rates and thus CT gene expression in cancer.

## Conclusion

Why some NSCLC cells express CT genes when others don’t, remains an interesting and unanswered question. We show a strong association between the normoactive allele of *MTHFR* 677 and CT gene expression in this study. This argues against the hypothesis of low level MTHFR activity leading to DNA hypomethylation, which in turn could lead to genome-wide hypomethylation and CT gene expression. However, due to the limited power of this study, we might have missed individual or cumulative effects of SNPs within other enzymes of the 1-carbon pathway on CT gene expression. SAM/SAH ratios for the tissues analyzed here were also unknown. Hence, we only contributed to, but did not resolve this interesting story, and hope future studies reveal the intricacies of the relation between CT gene expression and genetic variants of the 1-carbon pathway enzyme genes.

## Competing interests

The authors declare that they have no competing interest.

## Authors’ contributions

Overall study design: KMS, MG, ZK, YTC, AOG; patient recruitment and sample collection: NKA and YTC; genotyping experiments: KMS and ARB; principal component analysis and other *in silico* analyses: MI, OK and KMS; statistical analyses: MG, ZK, OK and KMS. All authors read and approved the final manuscript.

## Pre-publication history

The pre-publication history for this paper can be accessed here:

http://www.biomedcentral.com/1471-2350/14/97/prepub

## Supplementary Material

Additional file 1: Table S1 CT Gene Expression and Distribution of Clinical Parameters within NSCLC Patients. Click here for file

Additional file 2: Table S2 Genotypes of NSCLC Patients. Click here for file

Additional file 3: Table S3 Hardy-Weinberg Distributions of Single Nucleotide Polymorphisms in NSCLC Patients. Click here for file

Additional file 4: Figure S1 Principal component analysis based *in silico* clustering of AML. Tumor samples are shown ordered from the lowest to the highest first principal component (PC1) value. The 5 clusters generated by K-means clustering are indicated. Tumors with low, intermediate and high CT gene expression correspond to clusters 1-2, 3, and 4-5, respectively. Click here for file
