# Supplementary material for: Cancer-testis gene expression is associated with the methylenetetrahydrofolate reductase 677 C>T polymorphism in non-small cell lung carcinoma
Source: BMC Med Genet. 2013 Sep 24;14:97. doi: 10.1186/1471-2350-14-97 (PMC3849821; doi:10.1186/1471-2350-14-97)
Supplement: Additional file 1: Table S1 — CT Gene Expression and Distribution of Clinical Parameters within NSCLC Patients. [file 1471-2350-14-97-S1.doc]

SUPPLEMENTARY TABLE 1: CT Gene Expression and Distribution of Clinical Parameters within NSCLC Patients.

| # | Tumor (LU #) | NY-ESO-1 | LAGE-1 | MAGE-A1 | MAGE-A3 | MAGE-A4 | MAGE-A10 | CT-7 | SSX-2 | SSX-4 | Age | Sex | T Stage | Histology |
| --- | --- | --- | --- | --- | --- | --- | --- | --- | --- | --- | --- | --- | --- | --- |
| 1 | 31 | (+++) | (+/-) | (+) | (+++) | (+++) | N.D. | (+) | N.D. | N.D. | 57 | M | 2 | SQCC |
| 2 | 68 | (+++) | N.D. | (+) | (+) | N.D. | N.D. | (-) | N.D. | N.D. | 79 | M | 1 | Adeno |
| 3 | 87 | N.D. | (-) | (+++) | (+++) | N.D. | N.D. | (+++) | N.D. | N.D. | 65 | M | 1 | Adeno |
| 4 | 89 | (+++) | (+++) | (+++) | (+++) | (+/-) | (+) | (+/-) | N.D. | N.D. | 85 | F | 3 | Adeno |
| 5 | 111 | N.D. | (-) | (+++) | (+++) | (+++) | N.D. | (+++) | N.D. | N.D. | 55 | M | 4 | Lgcell |
| 6 | 131 | (+++) | (+/-) | (+) | (+++) | (+++) | N.D. | N.D. | N.D. | N.D. | 64 | M | 1 | SQCC |
| 7 | 168 | (+++) | (+++) | (+++) | (+++) | (+++) | N.D. | (+) | N.D. | N.D. | 70 | F | 1 | SQCC |
| 8 | 185 | (+++) | (+++) | (+++) | (+++) | (+/-) | (-) | (-) | (++) | (-) | 56 | F | 2 | NSCLC |
| 9 | 186 | (+++) | (+++) | N.D. | (+++) | N.D. | N.D. | N.D. | N.D. | N.D. | 61 | F | 3 | Adeno |
| 10 | 219 | (+) | (+) | (+++) | (+++) | (+++) | N.D. | (-) | (+/-) | (+++) | 54 | M | 2 | SQCC |
| 11 | 223 | (+++) | (+++) | N.D. | (+++) | (-) | N.D. | N.D. | N.D. | N.D. | 84 | M | 2 | SQCC |
| 12 | 649 | (+++) | (+/-) | (+++) | (+++) | (+/-) | (+++) | (-) | (-) | N.D. | 78 | M | 1 | AdenoBAC |
| 13 | 652 | (+++) | (+++) | (+++) | (+++) | (+++) | (+++) | (-) | (-) | N.D. | 75 | M | 2 | SQCC |
| 14 | 658 | (+/-) | (+++) | (+++) | (+++) | (+++) | (+/-) | N.D. | (+++) | N.D. | 80 | F | 3 | SQCC |
| 15 | 726 | (+++) | (+++) | (+++) | (+++) | (+++) | (+++) | N.D. | (++) | (+++) | 56 | F | 4 | Adeno |
| 16 | 736 | (+++) | (+++) | (+++) | (+++) | (+++) | (+++) | N.D. | (+/-) | (-) | 62 | F | 2 | Adeno |
| 17 | 739 | (+++) | (+++) | (+++) | (+++) | (+++) | (+++) | N.D. | (++) | (+++) | 60 | F | 2 | Lgcell |
| 18 | 745 | (+++) | (+++) | (+++) | (+++) | (+++) | (+/-) | N.D. | (++) | (-) | 63 | M | 2 | SQCC |
| 19 | 752 | (+++) | (+++) | (+++) | (+++) | (+++) | (+/-) | N.D. | (-) | (-) | U | U | U | U |
| 20 | 753 | (++) | (+/-) | (+++) | (+++) | (+++) | (+/-) | N.D. | N.D. | (-) | U | U | U | U |
| 21 | 759 | (+++) | (+++) | (+++) | (+++) | (+++) | (+/-) | N.D. | N.D. | (-) | U | U | U | U |
| 22 | 69 | (-) | (-) | (-) | (-) | N.D. | N.D. | (-) | N.D. | N.D. | 75 | M | 2 | BAC |
| 23 | 77 | (-) | (-) | (-) | (-) | N.D. | N.D. | (-) | N.D. | N.D. | 57 | M | 2 | SQCC |
| 24 | 88 | (-) | (-) | (-) | (-) | N.D. | N.D. | (-) | N.D. | N.D. | 78 | M | 1 | Adeno |
| 25 | 90 | (-) | (-) | (-) | (-) | N.D. | N.D. | (-) | N.D. | N.D. | 32 | M | 2 | Adeno |
| 26 | 108 | (-) | (-) | (-) | (-) | N.D. | N.D. | (-) | N.D. | N.D. | 74 | M | 1 | Adeno |
| 27 | 110 | (-) | (-) | (-) | (-) | N.D. | N.D. | (-) | N.D. | N.D. | 60 | M | 1 | Adeno |
| 28 | 112 | (-) | (-) | (-) | (-) | N.D. | N.D. | (-) | N.D. | N.D. | 72 | M | 2 | SQCC |
| 29 | 180 | (-) | (-) | (-) | (-) | N.D. | N.D. | (-) | (-) | (-) | 74 | F | 2 | Adeno |
| 30 | 183 | (-) | (-) | (-) | (-) | N.D. | N.D. | (-) | (-) | (-) | 64 | F | 1 | BAC |
| 31 | 191 | (-) | (-) | (-) | (-) | (-) | N.D. | (-) | N.D. | N.D. | 62 | F | 1 | Adeno |
| 32 | 221 | (-) | (-) | (-) | N.D. | (-) | N.D. | (-) | N.D. | N.D. | 62 | F | 2 | Adeno |
| 33 | 225 | (-) | (-) | (-) | (-) | (-) | N.D. | (-) | (-) | (-) | 79 | F | 2 | Adeno |
| 34 | 639 | (-) | (-) | (-) | (-) | (-) | (-) | (-) | N.D. | N.D. | 68 | F | 2 | AdenoBAC |
| 35 | 656 | (-) | (-) | (-) | (-) | (-) | (-) | N.D. | N.D. | N.D. | 83 | F | 1 | AdenoBAC |
| 36 | 670 | (-) | (-) | (-) | (-) | (-) | (-) | N.D. | N.D. | N.D. | 82 | F | 2 | AdenoBAC |
| 37 | 692 | (-) | (-) | (-) | (-) | (-) | (-) | N.D. | (-) | (-) | 56 | M | 1 | AdenoBAC |
| 38 | 693 | (-) | (-) | (-) | (-) | (-) | (-) | N.D. | (-) | (-) | U | U | U | U |
| 39 | 694 | (-) | (-) | (-) | (-) | (-) | (-) | N.D. | (-) | (-) | U | U | U | U |
| 40 | 698 | (-) | (-) | (-) | (-) | (-) | (-) | N.D. | (-) | (-) | 63 | F | 2 | Adeno |
| 41 | 706 | (-) | (-) | (-) | (-) | (-) | (-) | N.D. | (-) | (-) | 75 | M | 1 | Adeno |
| 42 | 707 | (-) | (-) | (-) | (-) | (-) | (-) | N.D. | (-) | (-) | 58 | F | 1 | Adeno |
| 43 | 713 | (-) | (-) | (-) | (-) | (-) | (-) | N.D. | (-) | (-) | 48 | F | 1 | AdenoBAC |
| 44 | 716 | (-) | (-) | (-) | (-) | (-) | (-) | N.D. | (-) | (-) | 80 | F | 1 | AdenoBAC |
| 45 | 718 | (-) | (-) | (-) | (-) | (-) | (-) | N.D. | (-) | (-) | 48 | M | 1 | AdenoBAC |
| 46 | 728 | (-) | (-) | (-) | (-) | (-) | (-) | N.D. | (-) | (-) | 72 | F | 2 | AdenoBAC |
| 47 | 748 | (-) | (-) | (-) | (-) | (-) | (-) | N.D. | (-) | (-) | 76 | M | 1 | AdenoBAC |
| 48 | 749 | (-) | (-) | (-) | (-) | (-) | (-) | N.D. | (-) | (-) | 78 | M | 1 | Adeno |
| 49 | 751 | (-) | (-) | (-) | (-) | (-) | (-) | N.D. | (-) | (-) | 70 | F | 2 | AdenoBAC |
| 50 | 763 | (-) | (-) | (-) | (-) | (-) | (-) | N.D. | (-) | (-) | U | U | U | U |

N.D.: not determined; U: unknown; M: male; F: female; SQCC: squamous cell carcinoma; Adeno: adenocarcinoma; BAC: bronchoalveolar carcinoma; AdenoBAC: adenocarcinoma with bronchoalveolar featuresl Lgcell: large cell carcinoma; NSCLC: other non-small cell carcinoma.
