# Supplementary material for: Cancer-testis gene expression is associated with the methylenetetrahydrofolate reductase 677 C>T polymorphism in non-small cell lung carcinoma
Source: BMC Med Genet. 2013 Sep 24;14:97. doi: 10.1186/1471-2350-14-97 (PMC3849821; doi:10.1186/1471-2350-14-97)
Supplement: Additional file 2: Table S2 — Genotypes of NSCLC Patients. [file 1471-2350-14-97-S2.doc]

SUPPLEMENTARY TABLE 2: Genotypes of NSCLC Patients.

| ***#*** | Tumor (LU #) | ***MTHFR 677 C>T***  (rs1801133) | ***MTHFR 1298 A>C***  (rs1801131) | ***MTR2756 A>G***  (rs1805087) | ***MTRR66 A>G***  (rs1801394) | ***RFC80 G>A***  (rs1051266) |
| --- | --- | --- | --- | --- | --- | --- |
| 1 | 31 | C/T | A/C | A/A | A/A | A/A |
| 2 | 68 | C/T | A/A | A/A | G/G | G/A |
| 3 | 87 | C/C | A/C | A/A | A/G | G/G |
| 4 | 89 | C/C | A/C | A/G | A/G | G/A |
| 5 | 111 | C/C | A/C | A/A | A/A | G/G |
| 6 | 131 | C/C | A/A | A/A | G/G | G/G |
| 7 | 168 | C/C | A/A | A/G | A/G | A/A |
| 8 | 185 | C/T | A/A | A/G | A/G | G/A |
| 9 | 186 | C/C | A/C | A/A | A/G | G/G |
| 10 | 219 | C/T | A/A | A/A | G/G | G/G |
| 11 | 223 | C/T | A/A | A/A | G/G | G/A |
| 12 | 649 | C/C | C/C | A/G | G/G | A/A |
| 13 | 652 | C/C | A/A | A/A | A/G | A/A |
| 14 | 658 | C/C | A/C | A/G | A/G | G/A |
| 15 | 726 | C/C | A/C | A/A | A/G | G/A |
| 16 | 736 | C/C | A/A | A/A | A/G | G/A |
| 17 | 739 | T/T | A/A | A/A | G/G | G/A |
| 18 | 745 | T/T | A/A | A/A | G/G | G/G |
| 19 | 752 | C/C | A/A | A/A | A/G | A/A |
| 20 | 753 | C/C | A/A | A/G | A/A | A/A |
| 21 | 759 | T/T | A/A | A/G | A/A | G/A |
| 22 | 69 | C/T | A/C | A/A | G/G | G/A |
| 23 | 77 | T/T | A/A | A/A | A/G | G/G |
| 24 | 88 | C/T | A/A | A/A | A/G | G/G |
| 25 | 90 | C/T | A/A | A/A | A/A | G/A |
| 26 | 108 | C/C | A/C | A/A | A/A | G/A |
| 27 | 110 | C/C | C/C | A/A | G/G | A/A |
| 28 | 112 | C/T | A/C | A/A | G/G | G/A |
| 29 | 180 | T/T | A/A | A/G | A/A | G/A |
| 30 | 183 | C/T | A/C | A/A | A/G | A/A |
| 31 | 191 | C/C | A/C | A/A | A/G | G/A |
| 32 | 221 | T/T | A/A | A/G | G/G | G/G |
| 33 | 225 | C/C | A/C | A/A | A/A | G/A |
| 34 | 639 | C/T | A/C | A/A | A/G | G/G |
| 35 | 656 | T/T | A/A | A/A | A/G | G/G |
| 36 | 670 | C/T | A/A | A/A | A/G | G/A |
| 37 | 692 | C/T | A/A | A/G | A/G | G/G |
| 38 | 693 | T/T | A/A | A/A | A/A | G/A |
| 39 | 694 | C/T | A/C | A/A | A/A | G/G |
| 40 | 698 | C/T | A/C | A/G | A/G | G/G |
| 41 | 706 | T/T | A/A | A/G | A/A | A/A |
| 42 | 707 | C/T | A/C | A/G | A/G | G/A |
| 43 | 713 | C/C | A/C | A/A | G/G | G/G |
| 44 | 716 | C/T | A/C | A/G | G/G | G/G |
| 45 | 718 | C/T | A/A | A/A | A/A | A/A |
| 46 | 728 | C/C | A/C | A/A | A/A | G/G |
| 47 | 748 | C/T | A/A | A/A | G/G | G/A |
| 48 | 749 | C/T | A/A | A/A | A/G | G/A |
| 49 | 751 | T/T | A/A | A/A | G/G | G/A |
| 50 | 763 | C/C | C/C | A/A | A/G | G/G |
