# Supplementary material for: Cancer-testis gene expression is associated with the methylenetetrahydrofolate reductase 677 C>T polymorphism in non-small cell lung carcinoma
Source: BMC Med Genet. 2013 Sep 24;14:97. doi: 10.1186/1471-2350-14-97 (PMC3849821; doi:10.1186/1471-2350-14-97)
Supplement: Additional file 3: Table S3 — Hardy-Weinberg Distributions of Single Nucleotide Polymorphisms in NSCLC Patients. [file 1471-2350-14-97-S3.doc]

**SUPPLEMENTARY TABLE 3: Hardy-Weinberg Distributions of Single Nucleotide Polymorphisms in NSCLC Patients.**

| ***Polymorphism*** | ***Genotype*** | ***Observed*** | ***Observed/ Expected*** | ***χ2*** | ***P**** |
| --- | --- | --- | --- | --- | --- |
| *MTHFR* 677 C>T (rs1801133) | CC | 20 | 1.1 | 1.389 | 0.24 |
|  | CT | 20 | 0.8 |  |  |
|  | TT | 10 | 1.3 |  |  |
|  |  |  |  |  |  |
| *MTHFR* 1298 A>C (rs1801131) | AA | 27 | 1 | 0.078 | 0.78 |
|  | AC | 20 | 1 |  |  |
|  | CC | 3 | 0.9 |  |  |
|  |  |  |  |  |  |
| *MTR*2756 A>G (rs1805087) | AA | 36 | 1 | 1.325 | 0.25 |
|  | AG | 14 | 1.2 |  |  |
|  | GG | 0 | - |  |  |
|  |  |  |  |  |  |
| *MTRR*66 A>G (rs1801394) | AA | 15 | 1.1 | 0.703 | 0.4 |
|  | GA | 22 | 0.9 |  |  |
|  | GG | 13 | 1.1 |  |  |
|  |  |  |  |  |  |
| *RFC*80 G>A (rs1051266) | GG | 18 | 1.1 | 0.469 | 0.49 |
|  | GA | 22 | 0.9 |  |  |
|  | AA | 10 | 1.1 |  |  |

* Chi-Square test (one degree of freedom).
