# Supplementary material for: Cancer-testis gene expression is associated with the methylenetetrahydrofolate reductase 677 C>T polymorphism in non-small cell lung carcinoma
Source: BMC Med Genet. 2013 Sep 24;14:97. doi: 10.1186/1471-2350-14-97 (PMC3849821; doi:10.1186/1471-2350-14-97)
Supplement: Additional file 4: Figure S1 — Principal component analysis based in silico clustering of AML. Tumor samples are shown ordered from the lowest to the highest first principal component (PC1) value. The 5 clusters generated by K-means clustering are indicated. Tumors with low, intermediate and high CT gene expression correspond to clusters 1-2, 3, and 4-5, respectively. [file 1471-2350-14-97-S4.docx]

**Supplementary Figure 1. Principal component analysis based *in silico* clustering of AML.** Tumor samples are shown ordered from the lowest to the highest first principal component (PC1) value. The 5 clusters generated by K-means clustering are indicated. Tumors with low, intermediate and high CT gene expression correspond to clusters 1-2, 3, and 4-5, respectively.
